# Anti-arthritic efficacy of geraniol and methotrexate via miRNA-driven NLRP3 inflammasome suppression in rat adjuvant-induced arthritis

**DOI:** 10.1007/s10787-026-02198-2

**Published:** 2026-04-16

**Authors:** Mennatallah A. Gowayed, Sarah A. Hassan, Maher A. Kamel, Maged W. Helmy, Samar O. El-Ganainy

**Affiliations:** 1 Department of Pharmacology and Therapeutics, Faculty of Pharmacy and Drug Manufacturing, Pharos University in Alexandria, Canal El Mahmoudia Street, Beside Green Plaza Complex, Alexandria, 21648 Egypt; 2 Department of Pathology, Faculty of Medicine, Alexandria University, Alexandria, Egypt; 3 Department of Biochemistry, Medical Research Institute, Alexandria University, Alexandria, Egypt; 4 Department of Pharmacology and Toxicology, Faculty of Pharmacy, Damanhour University, Damanhour, Egypt; 5 Department of Pharmacology, College of Pharmacy, Arab Academy for Science, Technology and Maritime Transport, Abu Kir Campus, Alexandria, 1029 Egypt

**Keywords:** Adjuvant-induced arthritis, Geraniol, Inflammasome, NLRP3, miR-124, miR-30a

## Abstract

**Objective:**

Geraniol (GO), a traditional plant-derived medicine, has demonstrated anti-osteoclastogenic properties that preserve bone integrity and inhibit bone resorption. This study aimed to evaluate the effect of GO in the adjuvant arthritis rat model, alone and combined with methotrexate (MX), with emphasis on miR-124 and miR-30a effect on the inflammasome (NLRP3)-related disease progression pathways.

**Methods:**

Fourteen days post-adjuvant injection, male Sprague–Dawley rats were treated with methotrexate (MX, 1 mg/kg/week), low-dose GO (100 mg/kg/day), high-dose GO (200 mg/kg/day), or a combination of MX and high-dose GO for 14 days. Arthritis progression was assessed using the arthrogram score and hind paw swelling. Tibiotarsal joint tissue was analyzed for inflammatory and autophagy markers, angiogenic factors, miR-124, and miR-30a. Radiological and histopathological examination of the ankle joint was performed, as well as immunohistochemistry for NLRP3.

**Results:**

Increased hind paw swelling and changes in radiological and histopathological characteristics confirmed the development of AA. Biochemical analysis of synovial tissue revealed decreased expression of miR-124 and miR-30a, accompanied by increased NLRP3 expression, as well as enhanced autophagy, angiogenesis, and inflammatory markers. Geraniol, in a dose-dependent manner, could reverse inflammatory parameters without any significant toxicological signs.

**Conclusion:**

These findings suggest that GO modulates the NLRP3 pathway through miRNA regulation. The synergistic effect of the GO and MX combination uncovers a novel therapeutic strategy in RA.

## Introduction

Geraniol (GO), a plant-derived monoterpene found in many essential oils known as *Geranium maculatum* L., has been widely used in traditional medicine for its wide range of pharmacological activities, including anti-inflammatory, antioxidant, antimicrobial, and neuroprotective activities (Ben Ammar 2023). Recently, GO displayed anti-inflammatory and anti-arthritic effects in different in vivo and in vitro models of rheumatoid arthritis (RA) (Malik et al. 2023; Naseer et al. 2023). Its anti-osteoclastogenic properties have retained bone integrity and diminished bone resorption (Malik et al. 2023). Such an effect has been associated with the complete suppression of nuclear factor kappa B (NF-kB) and the associated inflammatory pathways (Ben Ammar 2023; Malik et al. 2023).

Progression of RA is associated with damage to the articular cartilage, together with inflammation and hyperplasia of the synovium. Several immune cells and cytokines, including tumor necrosis factor α (TNF-α) and interleukin-1β (IL-1β), together with autoantibodies, mediate the breakdown of cartilage and bone (Alivernini et al. 2022). Recently, the nucleotide-binding oligomerization domain (NOD)-like receptor family pyrin domain containing 3 (NLRP3) inflammasome pathway is a main source of IL-1β by activating caspase-1 (Yin et al. 2022), also triggering the caspase-1-dependent pyroptosis (Mangan et al. 2018). Activated NLRP3 in synovial macrophages was associated with increased clinical arthritis severity and radiological bone destruction (Li et al. 2018a). Several NLRP3 inhibitors have been developed to halt RA progression. Inhibitors of the ROS/NF-κB/ NLRP3 inflammatory axis (Jing et al. 2021; Cao et al. 2022), as well as inhibitors of the mammalian target of rapamycin (mTOR) that induce autophagy-related inhibition of NLRP3, (Chen et al. 2019) have proven successful.

MicroRNAs (miRNAs) are among the regulators of the NLRP3 inflammasome at the post-transcriptional level (Zamani et al. 2020). Bioinformatic analysis and experimental studies have identified miR-30a as a significant negative regulator of NLRP3, reducing joint inflammation and attenuating bone damage in RA (Yang et al. 2021). Studies also highlighted the diagnostic value of miR-124 expression in synovial fluid and peripheral blood of RA patients (Wu et al. 2023; Nakamachi et al. 2009; Li et al. 2018a). Its activation was associated with decreased synovial proliferation, leucocyte infiltration, and synovial destruction in the adjuvant-induced arthritis model (Nakamachi et al. 2016), supposed to be mediated by inhibition of the NF-κB/NLRP3-associated proinflammatory response (Yang et al. 2022). Thus, miR-30a and miR-124 are good candidates for therapeutic use in RA.

To further elucidate the relationship between miR-30a/ miR-124 activation and inhibition of the NLRP3 signaling pathways in RA, this study investigated geraniol in low and high doses as a targeted therapeutic agent. Inflammatory, angiogenic, autophagic, and immunological markers were quantified to assess the full molecular pathway. Arthrogram scores, radiological and histopathological examinations were performed to assess anti-arthritic effects.

## Materials and methods

### Animals

Male Sprague–Dawley rats (160–200 g) were purchased from the animal house of Pharos University in Alexandria, Egypt. They remained under observation for one week before the study and had free access to food and water. Procedures were performed following the Institutional “Research Ethics Approval Committee” of the Faculty of Pharmacy, Pharos University in Alexandria, Egypt (No.01202101033020) and complying with ARRIVE guidelines and the National Institute of Health for the care and use of laboratory animals.

### Chemicals and drugs

Incomplete Freund’s adjuvant was purchased from Sigma Aldrich Co. (MO, USA), and mycobacterium butyricum was obtained from Difco Laboratories Co. (NJ, USA) to induce adjuvant arthritis. ELISA kits for TNF-α, NF-κBp65, vascular endothelial growth factor (VEGF), and IL-1β were acquired from Chongqing Biospes Co. (Cat. No. BEK1214, BYEK3040, BEK1228, and BEK1096, respectively, Chongqing, China), and for mTOR were acquired from LSBIO (Cat. No. LS-F17553, USA). The colorimetric kit for assessment of caspase-1 activity was obtained from ELabScience (Cat. No. E-CK-A381, USA). All RNA isolation and reverse transcription kits were purchased from Qiagen (Cat. No. 74104 and Cat. No. 205311, USA). MicroRNAs were assayed using TaqMan® MicroRNA assays (Cat. No. 4427975, ThermoFisher Scientific, USA). The monoclonal antibody for immunohistochemistry was bought from ABclonal Technology (A5652, ABclonal, USA). Methotrexate (MX) was purchased from Baoji Guokang Biotechnology Co., LTD, while Geraniol was purchased from Carl Roth (Karlsruhe, Germany) of purity ≥ 90%. 

### Induction of adjuvant arthritis (AA)

Rats were injected intradermally at the base of the tail with 0.1 ml suspension of heat-killed Mycobacterium butyricum in incomplete Freund’s adjuvant (12 mg/ml) (El-Refaie et al. 2023). Rats were then monitored for 13 days for the development of the AA model.

### Experimental design

Fourteen days after adjuvant injection, 48 rats were allocated into six groups (n = 8). Group 1, non-arthritic healthy control rats (NR), received a daily oral dose of saline. Group 2, adjuvant arthritic rats (AA), received a daily oral dose of saline. Group 3, AA rats were treated once weekly with methotrexate (MX), 1 mg/kg/week i.p. (El-Refaie et al. 2023). Group 4, AA rats were treated daily with a low dose of geraniol (GL) orally (100 mg/kg/day). Group 5, AA rats were treated daily with a high dose of geraniol (GH) orally (200 mg/kg/day). Group 6, AA rats were treated daily with GH orally (200 mg/kg/day) and once weekly with MX, 1 mg/kg/week i.p. (MG). Drugs were administered for 14 days (from day 14 to day 27 after induction).

### Assessment of arthritis progression

The hind paw swelling of the ankle (tibiotarsal joint) was measured using a vernier caliper on day 0 before the induction and then every other day until the end of the experiment (El-Refaie et al. 2023). The arthrogram 4-point scale scoring system was considered a measure of acute arthritis (0 = normal; 1 = slight edema of small digital joints; 2 = edema of the digital joints and footpad; 3 = gross edema of the entire footpad below the ankle or wrist; 4 = gross edema of entire footpad including the ankle joint or wrist joint) (El-Refaie et al. 2023). The score was assessed on days 0, 13, 19, 22, and 27.

### Radiological examination

On day 27, rats were anesthetized using i.p. ketamine/xylazine (80/5 mg/kg) and examined radiologically for any bone changes by plain X-ray. Rats were placed over a radiographic cassette containing standard X-ray film, 90 cm from the X-ray source, and exposed for 0.1 s to obtain lateral and oblique views. Three regions of interest (epiphysis, metaphysis, and diaphysis) were considered. The limb regions were manually cropped from the digitalized images. Radiographs were graded according to Table 1 and performed by two observers blinded to the study. For each hind paw, the maximum score was 21, and the mean score of both hind paws was calculated and presented.

**Table 1:** Radiographs scoring system

	Scores				
Periosteal reaction	0 (absent)	1 (mild)	2 (moderate)	3 (severe)	
Metaphyseal widening					
Osteolysis					
Bone deformation	0 (normal)	1 (minimal joint destruction)	2 (mild to moderate joint destruction)	3 (moderate joint destruction)	4 (marked to severe joint destruction with evident periarticular erosive changes)
Sequestrum formation	0 (absent)	1 (mild)	2 (severe)		
Joint effusion					
Soft tissue swelling	0 (normal)	1 (minimal soft tissue swelling)	2 (mild to moderate soft tissue swelling)	3 (moderate soft tissue swelling)	4 (marked to severe soft tissue swelling)

### Serum parameters

On day 28 of the study, rats were euthanized using an overdose of phenobarbital (200 mg/kg). Blood was collected from the posterior vena cava via laparotomy incision, and the serum levels of aspartate aminotransferase (AST), alanine aminotransferase (ALT), urea, and creatinine were determined spectrophotometrically using conventional kits.

### Tissue parameters

At the end of the study (day 28), the tibiotarsal joint of the right hind paw was detached and kept at − 80 °C till assay. The synovial tissue of the joint was isolated and divided into two aliquots. One aliquot was used for measuring tumor necrosis factor-alpha (TNF-α), nuclear factor kappa B (NF-κB), vascular endothelial growth factor (VEGF), interleukin-1β (IL-1β), mammalian target of rapamycin (mTOR), and caspase-1 activity using ELISA kits according to the manufacturer’s instructions. The second aliquot was used for total RNA extraction using miRNeasy kit according to the manufacturer’s instructions, then the reverse transcription of total RNA into cDNA was performed using QuantiTect Reverse Transcription Kit (Qiagen, Germany) according to the manufacturer’s instructions. Followed by the assessment of gene expression using qRT-PCR techniques.

### Measurement of gene expression in synovial tissues

The cDNA produced was used to quantify the joint expression of NLRP3, matrix metalloproteinase-9 (MMP-9), and Beclin-1 by Rotor-Gene Q qPCR utilizing QuantiTect SYBR Green PCR Master Mix. PCR amplification began with a denaturation phase for 10 min at 95 °C, followed by amplification of 40 cycles as follows: Denaturation (95 °C, 5 s), annealing (55 °C, 15 s), and extension (60 °C, 15 s). Table 2 presents the primers used in the experiment. Values of the threshold cycle (Ct) were determined by Rotor-Gene Q-Pure Detection (version 2.1.0). For each gene, the change in mRNA level in the samples was determined using the 2^−ΔΔCt^ method and normalized to the reference gene 18 s rRNA (Livak and Schmittgen 2001).

**Table 2:** Primer sequences of the investigated genes

Gene	Primer sequences
NLRP3	F: 5′- GTCCAGTGTGTTTTCCCAGAC -3′
	R: 5′- TTGAGAAGAGACCTCGGCAG -3′
MMP-9	F: 5′- TCGAAGGCGACCTCAAGTG -3′
	R: 5′- TTCGGTGTAGCTTTGGATCCA -3′
Beclin-1	F:5′-TTGGCCAATAAGATGGGTCTGAA-′3
	R: 5′- TGTCAGGGACTCCAGATACGAGTG-3′
18s rRNA	F: 5′- GTAACCCGTTGAACCCCATT -3′
	R: 5′- CAAGCTTATGACCCGCACTT -3′

### Synovial expression of miRNAs

The synovial expression of miR-124 and miR-30a was assayed using TaqMan® MicroRNA assay kit. Quantitative PCR began with an initial denaturation at 95 °C for 10 min and amplification via 45 cycles of PCR as follows: Denaturation at 95 °C for 5 s, annealing at 55 °C for 15 s, and then extension at 60 °C for 15 s. Amplification, data acquisition, and analysis were performed on the Rotor-Gene Q thermal cycler (Qiagen, USA). The values of the threshold cycle (Ct) were determined by Rotor-Gene Q-Pure Detection (version 2.1.0). The relative change in miR-124 and miR-30a in samples was determined using the 2-ΔΔCt method and normalized to the reference U6 as described previously (El-Khoury et al. 2016).

### Histopathological study

The left hind paw (tibiotarsal joint), liver, lung, and spleen were removed, washed with ice-cold saline, and fixed in 10% neutral buffered formalin for 24 h for further histopathological examination. Decalcification of hind paws was done using 5% formic acid. This was followed by routine processing of each specimen and embedding into paraffin blocks. Two non-serial 5-µm-thick sections were cut from each specimen and stained with hematoxylin–eosin (H&E) stain. Histopathological examination of hind paw joints from all groups was performed, and several histopathological parameters (synovial inflammation, synovial hyperplasia, synovial fibrosis, synovial angiogenesis, extension of pannus, cartilage erosion, and bone erosion) were assessed semi-quantitatively as described previously (Bendele et al. 1999; Douni et al. 2003; Brenner et al. 2005). An average score was taken from each paraffin block examined from each animal according to the recent guidelines (Hayer et al. 2021).

### Immunohistochemistry staining for NLRP3

Another 5-µm-thick sections were cut from the paraffin blocks of all the studied groups and were mounted on coated slides for subsequent immunohistochemical staining using NLRP3 antibody (A5652, ABclonal, USA) according to the manufacturer’s protocol. Positive NLRP3 staining was assessed as cytoplasmic staining in the synovial lining cells and /or the underlying tissues. The proportion of the immunostained cells was assessed semi-quantitatively using the following scale: 0 = no staining or a single positive cell, 1 =  < 10% positive cells, 2 = 11–50% positive cells, 3 = 50–80% positive cells and 4 ≥ 80% positive cells, and the staining intensity was evaluated based on the following scoring system: 0 = background color, 1 = pale yellow slightly higher than background color, 2 = brown, significantly higher than background color, 3 = tan. Finally, based on the sum of both scores, the expression of NLRP3 antibody was divided into four levels: (negative < 10% positive cells, regardless of the staining intensity, weak 3 points, moderate 4–5 points, strong 6–7 points). Five random fields were selected in each section, and an average score was calculated (Zhang et al. 2016).

### Statistical analysis

Data values are presented as means ± SD (n = 8). Data were analyzed using one-way analysis of variance (ANOVA), followed by a Tukey multiple comparison post hoc test. The differences were considered significant at *p* < 0.05. The graphs were drawn using the Prism computer program (GraphPad Software Inc., V8, San Diego, CA, USA). Two-way ANOVA was used to test for drug combination synergism or additive effective by considering each drug as independent factors then their combination and measuring the interaction between them (Slinker 1998).

## Results

### Effect on arthritis progression

**Fig. 1:**
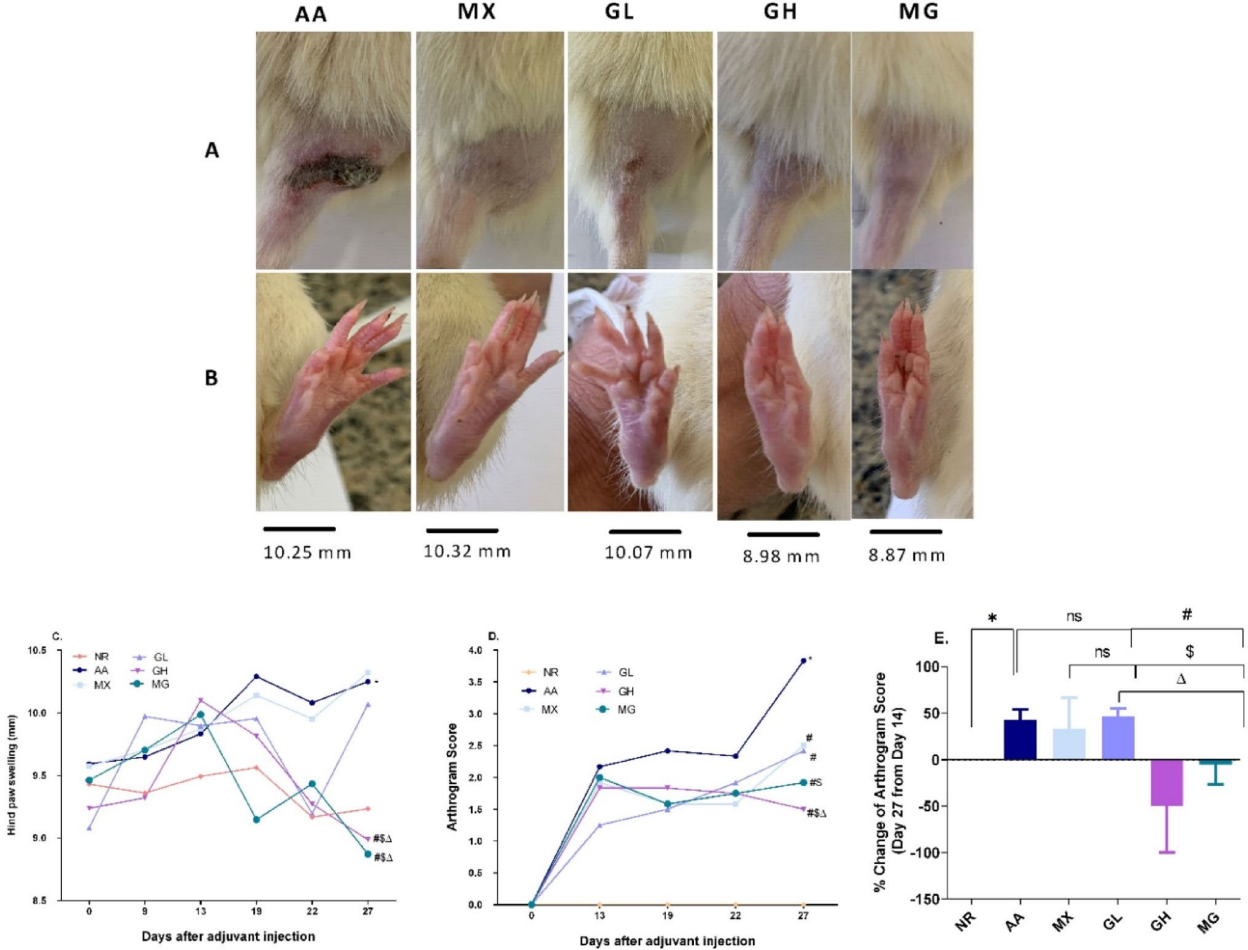
Evaluation of arthritis progression. **A** Representative photographs of the ulcer at the base of the tail, **B** Representative photographs of the hind paw swelling, **C** Hind paw swelling, **D** Arthrogram score, **E** % Change of Arthrogram Score. *p < 0.05 vs NR, #*p* < 0.05 vs AA, $ vs MX, ∆*p* < 0.05 vs GL, ℇ *p* < 0.05 vs GH; NR; normal control, AA; adjuvant arthritis, MX; methotrexate treated group, GL; low dose geraniol treated group, GH; high dose geraniol treated group, MG; MX + GH treated group. Data was analysed using one-way ANOVA followed by the Tukey Multiple Comparison Test at *p* < 0.05, and data is presented as mean ± SD

The hind paw swelling, and arthrogram score are considered indicators of inflammation and arthritic development. On day 13, before the start of the treatment, all rats reached a significant increase in hind paw diameter (mean 9.97 mm) and an arthrogram score of 2 (Fig. 1) compared to healthy controls (mean 9.43 mm and arthrogram score 0). On day 27, at the end of the treatment period, there was no obvious ulceration at the base of the tail in the GH and MG groups compared to the others (Fig. 1A). The hind paw swelling of GH and MG-treated rats has shown a significant reduction, reaching 8.98 mm and 8.87 mm, respectively, compared to AA rats (10.25 mm, *p* < *0.001*), MX-treated rats (10.32 mm, *p* < *0.001*) and GL-treated rats (10.07 mm, *p* < *0.001*), Fig. 1B, C. The arthrogram score (Fig. 1D) results also reflect the progression of arthritis from day 13 to day 27, where on day 27 only GH and MG rats showed significant differences compared to MX (*p* < *0.0001 and p* < *0.01*) and AA (*p* < *0.0001*), (Fig. 1E).

### Radiographic evaluation

**Fig. 2:**
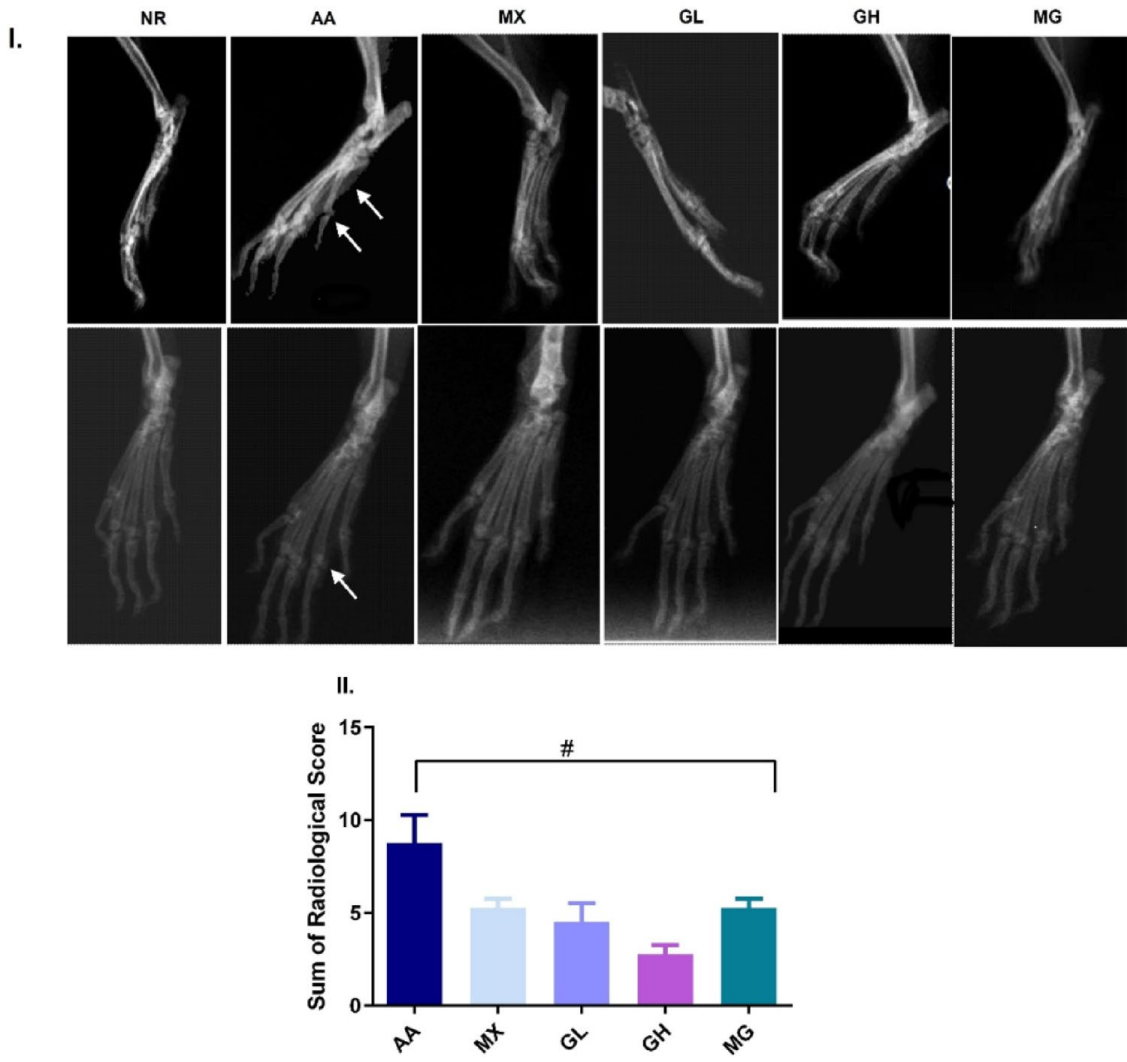
Radiographic changes in joints. **I** Representative images in lateral (upper panel) and oblique (lower panel) views. **II** Sum of radiological score. NR showing no pathological changes. AA joint showing severe inflammation with diminished joint space, marginal osteophytes, and bone matrix resorption. MX-treated joint showing evident signs of arthritis, with still noted bone resorption and tiny marginal osteophytes. GL-treated joint showing moderate inflammation with less reduction in joint space. GH -treated joints showing residual minimal osteoarthritic changes with no evidence of bone erosion, or soft tissue swelling, and preserved joint space. MG-treated joint showing mild signs of arthritis with evident bone resorption, tiny marginal osteophytes, and joint space narrowing. 1 = joint space, bone resorption and marginal osteophytes (if present), 2 = soft tissue swelling. #*p* < 0.05 vs AA, $ vs MX, ∆*p* < 0.05 vs GL, ℇ *p* < 0.05 vs GH; NR; normal control, AA; adjuvant arthritis, MX; methotrexate treated group, GL; low dose geraniol treated group, GH; high dose geraniol treated group, MG; MX + GH treated group. Data was analysed using one-way ANOVA followed by the Tukey Multiple Comparison Test and presented as mean ± SD

Radiographic examination of healthy control rats showed normal bone structure with intact joint space and no joint effusion (Fig. 2I). Erosive changes indicated radiographic bone deformities in AA rats, associated with new bone formation resulting in joint space narrowing and mild joint effusion (Fig. 2). All treated groups have shown enhanced joint microenvironment evident as a significant decrease in bone formation, osteolysis, and hence bone deformity (Table 3). However, it still revealed mild joint effusion and soft tissue swelling. Treatment with GL and GH expressed no joint space narrowing and no evident erosive changes, while treatment with MX alone or in combination with GH still revealed mild erosive changes pointed out within the joint and joint space narrowing (Fig. 2). The overall radiographic score (Fig. 2II) shows a significant enhancement of joint quality with all treatments examined; the lowest score was revealed in GH, however, non-significant different from other treatments.

**Table 3:** Effects of treatment on radiological examination

Experimental Groups	Periosteal reaction	Metaphyseal widening	Osteolysis	Bone deformation	Sequestrum formation	Joint effusion	Soft tissue swelling
NR	0 (0–0)	0 (0–0)	0 (0–0)	0 (0–0)	0 (0–0)	0 (0–0)	0 (0–0)
AA	2 (2–3)*	1 (1–2)*	1 (1–3)*	2 (2–3)*	0 (0–0)	1 (1–2)*	1 (1–2)*
MX	1 (1–2)*#	1 (1–2)*	0 (0–1)#	0 (0–1)#	0 (0–0)	1 (1–2)*	1 (1–2)*
GL	1 (1–2)*#	1 (1–2)*	0 (0–1)#	0 (0–1)#	0 (0–0)	1 (1–2)*	1 (1–2)*
GH	1 (1–2)*#	0 (0–1)#$ ∆	0 (0–1)#	0 (0–1)#	0 (0–0)	1 (1–2)*	1 (1–2)*
MG	1 (1–2)*#	1 (1–2)*ℇ	0 (0–1)#	1 (1–2)*∆ ℇ	0 (0–0)	1 (1–2)*	1 (1–2)*

Values are expressed as median with its minimum and maximum range (n = 8). #*p* < 0.05 vs AA, $ vs MX, ∆*p* < 0.05 vs GL, ℇ *p* < 0.05 vs GH; NR; normal control, AA; adjuvant arthritis, MX; methotrexate treated group, GL; low dose geraniol treated group, GH; high dose geraniol treated group, MG; MX + GH treated group

### Histopathological evaluation of the joint tissues

**Fig. 3:**
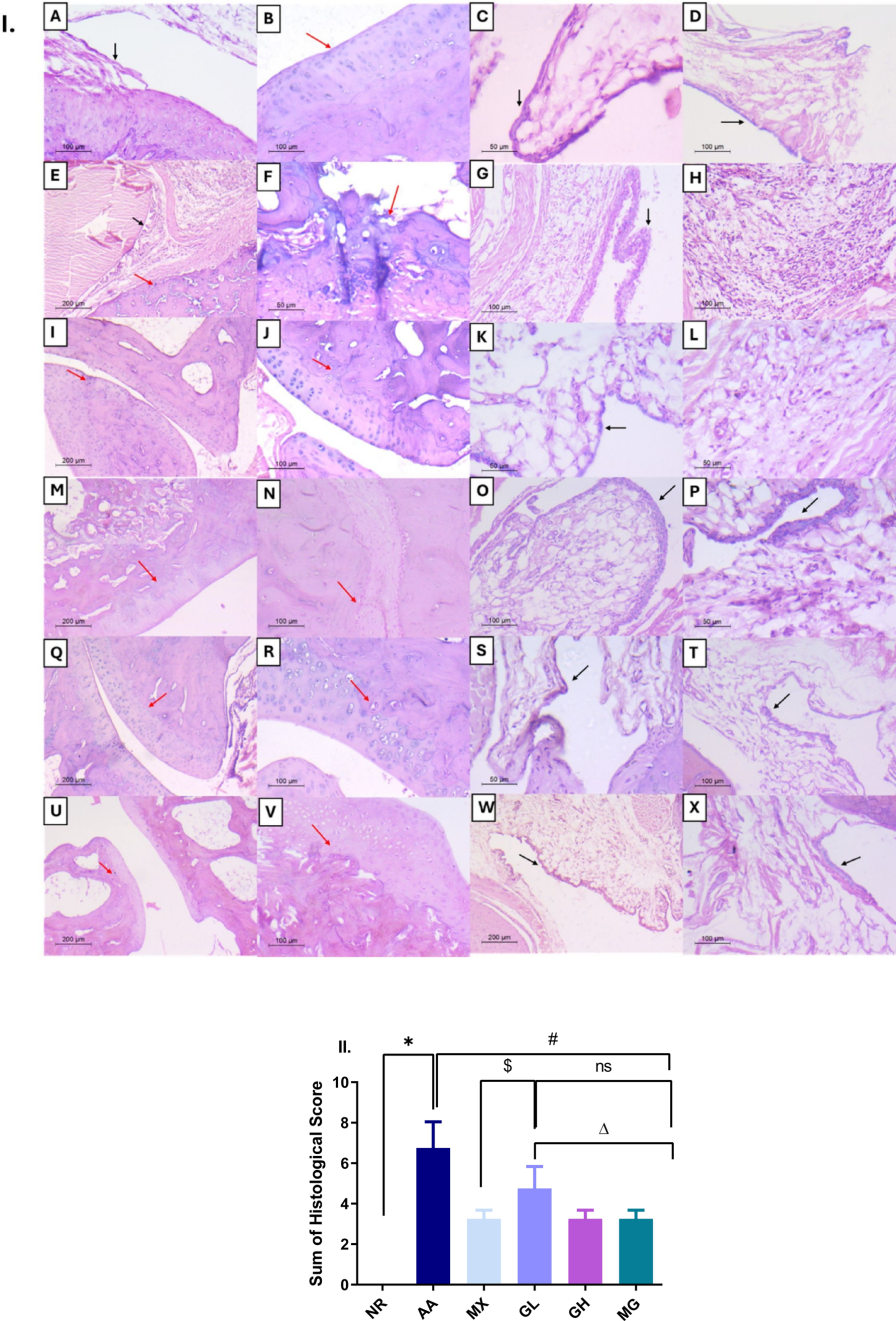
Histopathological evaluation of the joint tissues. I. Representative images of rat hind paw joint tissues in different study groups. NR group (A–D): **A** and **B** normal clear joint space and smooth intact articular cartilage with evident tidemark, **C** and **D** normal synovium lined by 1–2 layers of cells, the subintima lacked inflammation and showed normal vasculature and connective tissue. AA group (E–H): **E** and **F** narrowing of joint space, pannus formation, and irregular eroded articular cartilage and subchondral bone with lost tidemark were noted, **G** and **H** hyperplastic synovium, the subintima showed edema, dense mononuclear inflammatory infiltrate with increased vascularity. MX-treated group (I-L): **I** and **J** clear joint space with near normal articular cartilage showing minor irregularities and preserved tidemark was seen, **K** and **L** restoration of the normal synovium with reduction of the inflammation at the subintimal layer. GL-treated group (M-P): **M** and **N** restoration of normal articular cartilage architecture with normal zonation, only minor irregularities were noted (N). **O** and **P** the synovium was mildly hyperplastic in some cases, with a reduction of the inflammation at the subintimal layer (P). The GH-treated group (Q-T) showed more improvement, **Q** and **R** restoration of the normal articular cartilage architecture with preserved zones and tide mark. **S** and **T** normal synovial lining was observed, and the subintima lacked inflammation. MG-treated group (U–X), **U** and **V** comparable results were achieved, clear joint space with normal articular cartilage architecture, zones, and tidemark. **W** and **X** normal synovial lining with no inflammation and normal vascularity of the subintimal layer (H&E, E, I, M, Q, U and W: × 100-A, B, D, G, H, J, N, O, R, T, V and X: × 200- C, F, K, L, P and S: × 400). Red arrows: articular cartilage, black arrows: synovium. **II** The sum of all histological scores. #*p* < 0.05 vs AA, $ vs MX, ∆*p* < 0.05 vs GL, ℇ *p* < 0.05 vs GH; NR; normal control, AA; adjuvant arthritis, MX; methotrexate treated group, GL; low dose geraniol treated group, GH; high dose geraniol treated group, MG; MX + GH treated group. Data was analyzed using one-way ANOVA followed by Tukey’s test and presented as mean ± SD

Microscopic examination of the hind paw joints of NR revealed normal joint tissues showing clear joint space, intact smooth articular cartilage with normal zones, and evident tidemark. The underlying bone was intact and showed no erosion. The synovium was lined by one to two layers of synovial cells, and the subintima showed no inflammation, with normal vasculature and connective tissue (Fig. 3A-D). Examination of AA joints revealed narrowing of the joint space and obliteration by fibrous tissue. The articular cartilage showed erosion and irregularities and was occasionally thinned out in areas. Some of the chondrocyte lacunae were empty, and others showed pyknotic nuclei. The tide mark was absent, and the zonation was occasionally lost. The underlying subchondral bone showed cracks and exhibited mild osteoporotic changes.

Hyperplastic synovium was noted with occasional papillae formation, the subintima showed edema, increased vascularity, and a dense mononuclear inflammatory infiltrate formed of macrophages and lymphocytes. (Fig. 3E–H).

The MX-treated joints revealed amelioration of the pathological changes noted in the AA group. The articular cartilage was preserved, but focal minor irregularities were still noted. The tide mark was present but was occasionally interrupted. The synovium was not hyperplastic; however, focal small papillae were occasionally seen with a reduction of the inflammation seen at the subintimal layer. Moreover, the underlying bone was preserved (Fig. 3I–L).

Administration of GL resulted in comparable results to MX treatment, with amelioration of the changes seen in the AA model. Minor irregularities were occasionally noted within the articular cartilage. A focal interruption of the tide mark was detected. The synovium was not hyperplastic in most cases, with focal small papillae formation. Reduction of the inflammation at the subintimal layer was also noted (Fig. 3M–P). On the other hand, the group that received GH showed a more significant improvement. The articular cartilage’s normal architecture was restored, with preserved zonation and tide mark, and intact subchondral bone. The subintima lacked inflammation in most cases (Fig. 3Q–T).

Co-administration of MX with GL (MG) resulted in near normal joint appearance with comparable results to high-dose geraniol alone. Clear joint space, smooth intact articular cartilage, with preserved tide marks and zones. The subchondral bone was intact. The synovium appeared within normal limits without inflammation in the subintimal layer (Fig. 3U–X). The sum of all histological scores is presented in Fig. 3II.

### Effect of geraniol or methotrexate, or combined treatment on expression levels of miR-124, miR-30a, NLRP3, Beclin-1, MMP-9

**Fig. 4:**
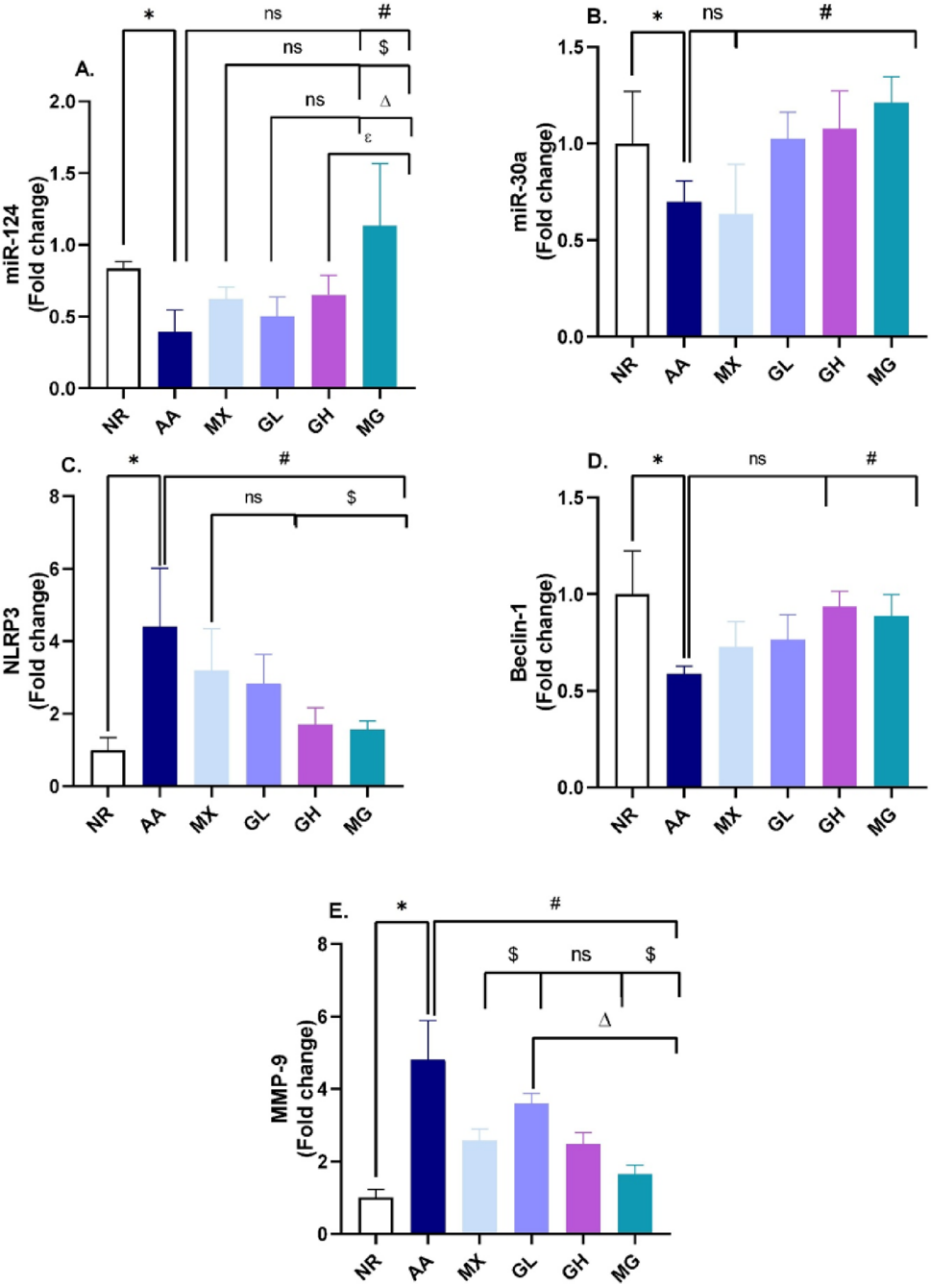
Effect of Geraniol, methotrexate, or combined treatment on expression levels of **A** miR-124, **B** miR-30a, **C** NLPR3, **D** Beclin-1, **E** MMP-9. *p < 0.05 vs NR, #*p* < 0.05 vs AA, $ vs MX, ∆*p* < 0.05 vs GL, ℇ *p* < 0.05 vs GH; NR; normal control, AA; adjuvant arthritis, MX; methotrexate treated group (1 mg/kg/week), GL; low dose geraniol treated group (100 mg/kg), GH; high dose geraniol treated group (200 mg/kg), MG; MX + GL treated group. Data was analyzed using one-way ANOVA followed by Tukey’s test and presented as mean ± SD

Assessment of miR-124 expression revealed a significant decline in its level in the adjuvant arthritis model. Treatment with high-dose geraniol (200 mg/kg) alone and combined with methotrexate was able to upregulate miR-124 expression levels (*p* < 0.05), showing only a significant effect in the combined treated group. On the other hand, miR-30a was significantly downregulated following the induction of rheumatoid arthritis in rats compared to the control group. Both doses of geraniol and combined treatment were able to restore their expression level in a significant manner, as shown in Fig. 4A, B.

NLRP3 expression levels showed an abrupt upregulation, reaching more than a fourfold increase in the adjuvant arthritic model (*p* < 0.05). The geraniol-treated group exhibited a significant dose-dependent downregulation of NLRP3 expression, as was also manifested in the combination group, with no significant difference among groups (Fig. 4C).

As a marker of autophagy, Beclin-1 expression was measured following rheumatoid arthritis induction. Results showed a 50% decline in its level compared to the control group (*p* < 0.05). Combined geraniol and methotrexate were the only treatments showing significant restoration of Beclin-1 expression levels (Fig. 4D).

Following adjuvant arthritis induction, MMP-9 expression was significantly upregulated concerning control rats. Both MX, GL, and GH treatments were able to suppress the expression compared to the AA group, while the MX combined treatment showed the lowest level of MMP-9 in the joint (*p* < 0.05), Fig. 4E.

### Evaluation of the effect of geraniol, methotrexate, or combined treatment on protein levels of TNF-α, NF-κB, VEGF, IL-1β, mTOR, and caspase-1

**Fig. 5:**
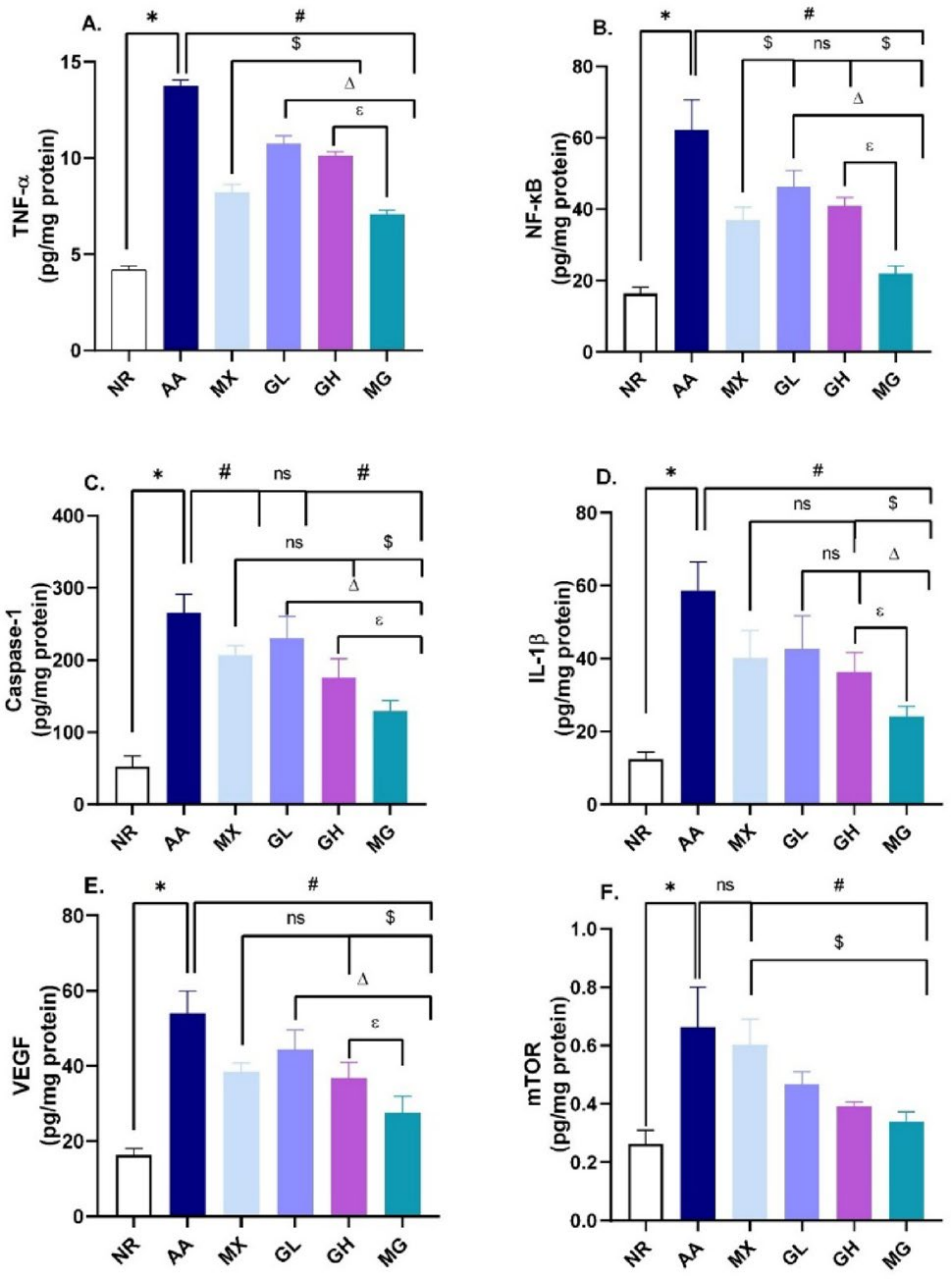
Effect of Geraniol, methotrexate, or combined treatment on protein levels of **A** TNF-α, **B** NF-κB, **C** Caspase-1 VEGF, **D** IL-1β, **E** VEGF, **F** mTOR. *p < 0.05 vs NR, #*p* < 0.05 vs AA, $ vs MX, ∆*p* < 0.05 vs GL, ℇ *p* < 0.05 vs GH; NR; normal control, AA; adjuvant arthritis, MX; methotrexate treated group (1 mg/kg/week), GL; low dose geraniol treated group (100 mg/kg), GH; high dose geraniol treated group (200 mg/kg), MG; MX + GL treated group. Data was analyzed using one-way ANOVA followed by Tukey’s test and presented as mean ± SD.

Determination of TNF-α content showed a threefold increase in its content in the AA group compared to control rats (*p* < 0.05). Although all treatments suppressed TNF-α levels, methotrexate alone or combined with geraniol induced the most prominent suppression among all groups. Parallel results were obtained for the downstream NF-κB, where combined geraniol and methotrexate treatment exhibited nearly normal levels of NF-κB, Fig. 5A, B.

In addition, IL-1β and its activator, caspase-1, exhibited a prominent rise following arthritis induction in the control group. Methotrexate, high-dose geraniol, and their combination were able to significantly suppress this rise, where the combined treatment showed the lowest levels of both IL-1β and caspase-1, Fig. 5C, D.

Concerning VEGF, its level was notably increased in AA compared to normal rats (*p* < 0.05). Geraniol induced a dose-dependent decline in VEGF tissue content, while combined treatment with MX possessed the superior effect. On the other hand, all treatments equally reversed the increase in mTOR tissue levels following adjuvant arthritis induction (*p* < 0.05). Results are shown in Fig. 5E, F.

### Evaluation of NLRP3 immunohistochemical staining in joint tissues

**Fig. 6:**
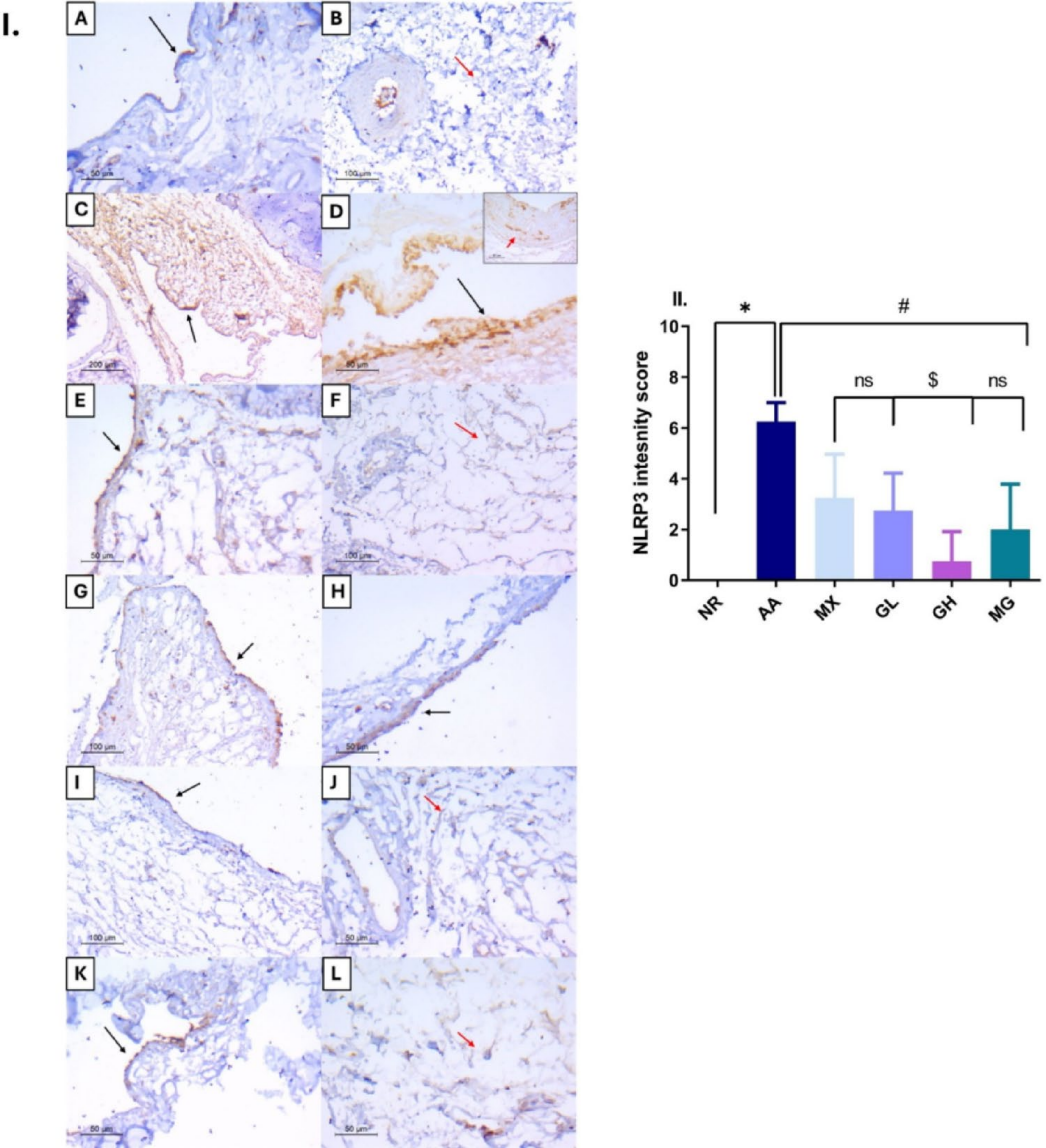
NLRP3 protein expression in hind paw joint tissues in different study groups. I. Representative images of NLRP3 immunostaining. **A** and **B** The NR group showed weak to absent staining for NLRP3 in the synovium and underlying tissues. **C** and **D** The AA group exhibited strong cytoplasmic staining in the synovium and the underlying stromal and mononuclear inflammatory cells (Figure D- inset). **E** and **F** The MX-treated group showed moderate NLPR3 expression. **G** and **H** The GL-treated group exhibited weak to moderate NLRP3 expression. **I** and **J** The GH-treated group exhibited the lowest NLRP3 protein expression scores. **K** and **L** The MG group showed moderate NLRP3 staining. (NLRP3 IHC- B, F, G and I, × 200- C, × 100- A, D, D-inset, E, F, H, J, K and L, × 400), black arrows = synovium, red arrows = underlying tissues. II. The NLRP3 intensity scores. #*p* < 0.05 vs AA, $ vs MX, ∆*p* < 0.05 vs GL, ℇ *p* < 0.05 vs GH; NR; normal control, AA; adjuvant arthritis, MX; methotrexate treated group, GL; low dose geraniol treated group, GH; high dose geraniol treated group, MG; MX + GH treated group. Data was analyzed using one-way ANOVA followed by Tukey’s test and presented as mean ± SD

Examination of NR-hind paw joint tissues showed weak cytoplasmic staining in the synovial cells and occasional weak staining observed in the sub-synovial tissue, with negative total NLRP3 scores (Fig. 6A-B). In contrast, sections from the AA arthritis group exhibited moderate to strong cytoplasmic staining in the synovial cells and the underlying subsynovial tissues, which was detected in the stromal cells and the mononuclear inflammatory cells (Fig. 6C, D). Administration of MX resulted in a moderate NLRP3 expression with a mild decrease in both the intensity and the proportion of positive cells in the joint tissues (Fig. 6E, F). On the other hand, the GL-treated group showed less staining intensity and proportion of positive cells compared to MX (Fig. 6G, H). However, the lowest total scores observed were in the GH-treated group (Fig. 6I, J). Interestingly, combining GH with MX resulted in higher NLRP3 total expression scores than GH alone (Fig. 6K–L). The NLRP3 total intensity scores showed that all treatments significantly suppressed inflammasome activation, as summarized in Fig. 6II.

### Histopathological evaluation of liver, lung, and spleen tissues

**Fig. 7:**
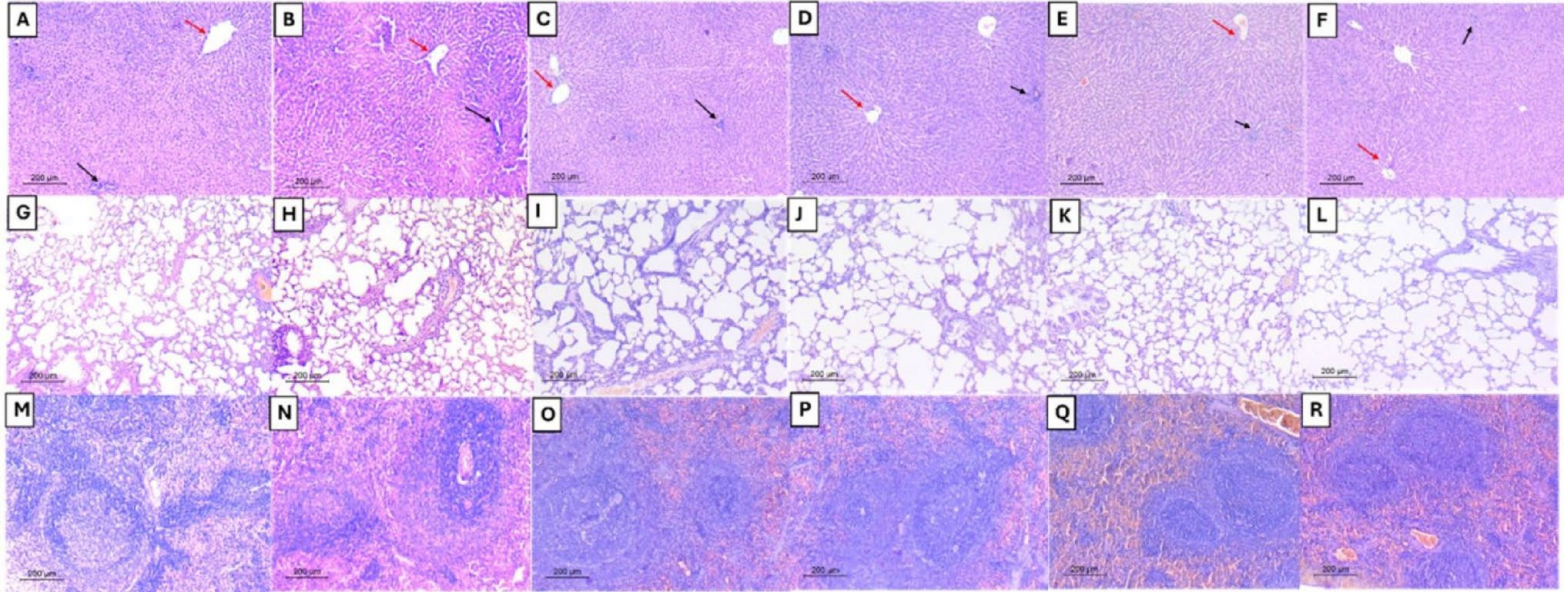
Histopathological evaluation of liver, lung, and spleen. Liver sections of the NR group **A** revealed normal hepatic architecture showing radiating cords of hepatocytes, normal central veins, and portal tracts. The AA group **B** revealed similar normal liver architecture and hepatocyte morphology. The MX-treated group **C** revealed preserved normal hepatic architecture and hepatocyte morphology. The GL-treated group **D** revealed preserved liver architecture and hepatocyte morphology. Similarly, the GH-treated group **E** revealed hepatic tissue within normal limits. The MG-group **F** revealed comparable results. **G**–**L** Lung sections examination revealed normal lung histology in the AA group, and all treated groups showed the same normal appearance. G: NR group, H: AA group, I: MX-treated group, J: GL-treated group, K: GH-treated group, L: MG-treated group. (M-R) Spleen sections examination revealed normal splenic white pulp showing lymphoid follicles with evident zonation and central arterioles, surrounded by red pulp showing splenic sinusoids in the NR group, AA group, and all treated groups. (M: NR, N: AA group, O: MX-treated group, P: GL-treated group, Q: GH-treated group, and R: MG-treated group, (H&E, A-R, × 100, red arrows = central veins and black arrows = portal tracts)

Histopathological examination of the liver tissues in the treated study groups revealed normal hepatic architecture, normal central veins, and portal triads. No sinusoidal dilatation, lobular or portal inflammation was detected. The hepatocytes were normal and showed no swelling or degeneration (Fig. 7A–F). Similar results were obtained following examination of the lung tissues from all the studied groups (Fig. 7G–L). The alveoli were pathologically unremarkable with very minimal interstitial inflammation and no congestion of the septal capillaries. No intra-alveolar exudate was noted. Normal bronchioles were seen. Sections of splenic tissues from all the studied groups were also within normal limits (Fig. 7 M–R). Normal red and white pulps were seen. The white pulp showed lymphoid follicles with normal zonation and central arterioles.

### Evaluation of different treatments on liver and kidney functions

Serum liver transaminases increased in a significant pattern following adjuvant arthritis induction. Similarly, AST was significantly high in the MX-treated group, comparable to the NR group. Geraniol, in both low and high doses, succeeded in suppressing transaminase, but combined with MX only significantly affected ALT level (*p* < 0.05). On the other hand, urea was significantly higher in AA compared to NR. A slight, non-significant decline was observed in the Geraniol and combined treatment groups. Serum creatinine was significantly raised following arthritis induction, an effect that was significantly reversed only in the GH group. Results are shown in Table 4.

**Table 4:** Effect of Geraniol, methotrexate, or combined treatment on serum levels of AST, ALT, Urea, and Creatinine

Group	Parameter			
	AST (U/L)	ALT (U/L)	Urea (mg/dL)	Creatinine (mg/dL)
NR	124.5 ± 1.86	40 ± 3.46	28.5 ± 1.94	0.59 ± 0.013
AA	165.8 ± 3.37*	70.5 ± 2.40*	36.8 ± 2.10*	0.74 ± 0.037*
MX	161 ± 12.53*	57 ± 2.38#	34.3 ± 2.24	0.83 ± 0.055
GL	105.5 ± 5.43#$Δ	50 ± 1.70#Δ	32.8 ± 1.01	0.72 ± 0.020
GH	86.50 ± 3.86#$ε	69.67 ± 2.00$ε	31.78 ± 1.18	0.57 ± 0.010#$ε
MG	143.5 ± 5.27	49 ± 3.75#	31.5 ± 2.46	0.79 ± 0.033

*p < 0.05 vs NR, #*p* < 0.05 vs AA, $ vs MX, ∆*p* < 0.05 vs GL, ℇ *p* < 0.05 vs GH; NR; normal control, AA; adjuvant arthritis, MX; methotrexate treated group (1 mg/kg), GL; low dose geraniol treated group (100 mg/kg), GH; high dose geraniol treated group (200 mg/kg), MG; MX + GL treated group. Data was analyzed using one-way ANOVA followed by Tukey’s test and presented as mean ± SD

### Evaluation of drug combination synergism

To evaluate the effect of combining MX and GR, Two-Way ANOVA was performed. The results revealed a synergistic interaction between the two drugs regarding arthrogram score, radiological and histopathological score (*p* < 0.05). On the level of molecular biomarkers, MX and GR combinations also exhibited significant synergism on the protein level of NLRP3, TNF and MMP9. In contrast, all other parameters demonstrated an additive effect of the drugs combination.

## Discussion

Inflammasome activation is crucial in regulating inflammation and immune response. Among inflammasomes, NLRP3 plays a key role in both innate and adaptive immunity. Despite its role in controlling infection, once overactivated, it leads to host tissue damage and contributes to inflammation-driven pathological states (Li et al. 2020). Recently, NLRP3 has been identified as a checkpoint in innate immunity that drives adaptive immunity to lose immunological self-control, leading to autoimmune diseases (Zhang et al. 2021). In the present work, adjuvant arthritis induced rats showed an abrupt increase in both expression and protein level of NLRP3, as previously reported in this model (Zhou et al. 2023; Ding et al. 2016). Stimulation of NLRP3 expression is regulated via various receptors, including the TNF-α receptor. Once bound to its receptor, TNF-α activates NF-κB production, which subsequently upregulates NLRP3 expression. Under such conditions, the canonical pathway is initiated, activating caspase-1, which in turn cleaves Interleukin-1β and IL-18 into their active form, inducing both inflammation and pyroptosis (Jiang et al. 2022). In line with this pathway, we observed a significant increase in protein levels of upstream mediators TNF-α and NF-κB, as well as downstream mediators including caspase-1 and IL-1β protein levels in the rat rheumatoid model.

Treatment with geraniol suppressed NLRP3 expression and protein level in a dose-dependent manner, with the high dose exceeding the methotrexate effect. Both TNF-α and NF-κB levels were reduced almost equally in MX and geraniol doses. However, combined MX and GR treatment showed a superior effect over MX alone. Similar results were observed with caspase-1 and IL-1β levels, which exhibited the lowest levels in combined treatment among all groups. Recently, Malik et al. reported a decline in TNF-α, NF-κB, and IL-1β expression following geraniol treatment (Malik et al. 2023). However, the study was limited to assessing inflammatory mediators without revealing the exact effector pathway.

Additional regulators of NLRP3 expression are non-coding microRNAs. During the transcriptionally active state, NLRP3 translation can be modulated by microRNAs via their binding to untranslated regions (Tezcan et al. 2019). Among various miRNAs, miR-30a has been reported to negatively regulate NLRP3 in rheumatoid arthritis. Yang et al. (Yang et al. 2021) showed that intraarticular injection of miR-30a inhibited NLRP3 inflammasome, halting joint inflammation and bone degeneration. This was via direct binding to the 3’ untranslated region of NLRP3. In the present work, adjuvant arthritis was accompanied by more than 30% decline in miR-30a expression. Only geraniol, in both doses and combined with methotrexate, was able to restore its expression level to normal values. Similarly, the downregulated miR-124 expression following arthritis induction was restored, but only significantly with combined methotrexate and geraniol treatment. It was previously shown that miR-124 could suppress NLRP3 expression, inhibiting microglial activation and neuroinflammation (Yang et al. 2022). Recently, the role of miR-124 in inhibiting inflammasomes in immune diseases has been described by Zhao et al. (Zhao et al. 2023) in an atopic dermatitis model. However, this study is the first to highlight its role in rheumatoid NLRP3-driven pathway as well as the effect of geraniol on their expression.

The mTOR signaling pathway has been known to integrate a wide spectrum of cellular and metabolic mediators that shape immune responses (Weichhart et al. 2015). It possesses a role in augmenting the inflammation process via stimulating multiple inflammatory mediators. Cosin-Roger et al. (Cosin-Roger et al. 2017) first identified NLRP3 inflammasome as a binding target of mTOR. In autoimmune diseases, mTOR positively regulates NLRP3, probably via glycolysis activation(Weichhart et al. 2015) and ROS production (Li et al. 2018b), increasing activation of pro-IL-1β. In the current study, mTOR was doubled in adjuvant arthritis rats. An effect that was reversed only by geraniol, either alone or combined with methotrexate. Additionally, mTOR is known to negatively regulate autophagy via phosphorylation, suppressing autophagic mediators (Ganley et al. 2009).

In harmony with the aforementioned pathway, stimulation of autophagy markers can lead to NLRP3 capture and degradation via autophagosomes. Also, autophagosomes curtail NLRP3 activation via reducing ROS generation (Shi et al. 2012; Biasizzo and Kopitar-Jerala 2020), highlighting the association between mTOR/Autophagy/NLRP3. Beclin-1, an autophagic marker favouring autophagosome formation, can interact with NLRP3 (Biasizzo and Kopitar-Jerala 2020), and its inhibition results in caspase-1 and IL-1β activation (Nakahira et al. 2011). Arab et al. (Arab et al. 2021) have documented that increasing autophagic flux expressed by Beclin-1 upregulation is mediated via mTOR inhibition, which in turn suppresses NLRP3 activation. This was in line with our results, where GO in high dose, alone or combined with MX, was able to restore Beclin-1 expression, which was associated with mTOR and NLRP3 suppression.

In line with the previous findings, adjuvant arthritis rats showed an upsurge in the expression of MMP-9 compared to controls. MMPs are protease enzymes that are expressed in chondrocytes and contribute to the pathogenesis of rheumatoid arthritis. Once activated, they induce extracellular matrix and joint destruction and stimulate various cell mediators, promoting an inflammatory cascade (Bian et al. 2023). Activation of the inflammasome pathway NLRP3 has been linked to upregulation of MMP-9 (Unamuno et al. 2021; Robert et al. 2019). Jia et al. (Jia et al. 2024) had found that NLRP3 inhibitor MCC950 induced MMP-9 reduction. In addition, deletion of the NLRP3 gene in mice resulted in suppressed MMP-9 and IL-1β expression and protein level damping inflammatory cascade (Shimizu et al. 2019). Furthermore, MMP-9 was identified as a downstream target of caspase-1 activation, which was linked to direct stimulation of MMP-9 (Jia et al. 2024; Leal et al. 2025). Together, these findings suggest a direct causal relation between the NLRP3 pathway and MMP-9 regulation. Overproduction of MMP-9 was also related to upregulated damage-associated molecular patterns, which are known to activate NLRP3, generating a vicious cycle of immune-related cell damage (Bian et al. 2023). Both Methotrexate and geraniol were able to induce a 50% reduction of MMP-9, while their combination presented near normal value. Earlier, geraniol has been reported to suppress MMP-9 in myocardial infarction (Zou et al. 2022) and osteoarthritis (Wu et al. 2020), but for the first time in autoimmune rheumatoid arthritis.

Activation of the NLRP3 inflammasome pathway is closely connected to abnormal neovascularization with overproduction of angiogenic factors, including VEGF (Ding et al. 2022). Knocking down of NLRP3 gene was accompanied by reduced inflammasome activation, impaired angiogenesis, and reduced VEGF (Chai et al. 2020; Zhang et al. 2023). In Rheumatoid pathogenesis, VEGF contributes to the inflammatory environment, osteoclast activation, and bone destruction. Targeting VEGF delays disease onset and synovial inflammation in mice (Lu et al. 2000; Afuwape et al. 2003). In the current study, geraniol, dose dependently, abrogated the rise in VEGF following arthritis induction, while combined with methotrexate showed the lowest VEGF protein level. Geraniol has previously been reported to possess multiple anti-angiogenetic effects (Wittig et al. 2015), but has never been explored in rheumatoid therapy.

In harmony with former molecular findings, the arthrogram score has been ameliorated among groups showing the best results with geraniol high dose alone or combined with methotrexate. Histopathological score summation, as well as radiological score, also showed pronounced improvement.

It is worth mentioning that both liver and kidney function were significantly impaired in adjuvant arthritis rats. Geraniol showed improved AST and ALT and nearly normal urea and creatinine serum levels, adding to its therapeutic potential in rheumatoid arthritis.

The co-administration of Geraniol (GR) and methotrexate (MX) demonstrated a synergistic effect on arthrogram score, radiological and histological changes. Such synergy on tissue levels was comported by significant reduction of inflammatory markers such as inflammasome NLRP3, TNF-α and MMP3. These findings showed that GR and MX combination synergistically alleviated arthritis features, in part by modulating inflammasome pathway. However, further studied are warranted to elucidate additional molecular pathways that may contribute to the enhanced therapeutic outcomes observed in this experimental model.

A potential limitation of this study is the use of two dose levels for the MX and GR combination. While these doses were sufficient to demonstrate potential synergistic effect, future investigations utilizing a wider dose–response gradient may provide additional insights into the potency and saturation kinetics of this co-therapy.

In conclusion, this study highlights the therapeutic potential of geraniol in alleviating adjuvant-induced arthritis in rats, as demonstrated by arthrograms, radiological imaging, and histopathological analysis. Geraniol exerts its anti-arthritic effects primarily through the inhibition of NLRP3 inflammasome activation, achieved by upregulating its upstream receptor regulator microRNAs, notably miR-30a and miR-124. This inhibition was accompanied by the downregulation of key inflammatory, immunological, angiogenic, and autophagic mediators, underscoring geraniol’s multi-targeted mechanism of action. Moreover, when administered in combination with methotrexate, geraniol enhanced molecular efficacy while also improving renal and hepatic function. Collectively, these findings support the potential of geraniol as a promising adjunctive agent in the treatment of rheumatoid arthritis.

## Data Availability

All data generated or analyzed during this study are included in this published article.

## Abbreviations

AA: Adjuvant arthritis
GH: High dose of geraniol
GL: Low dose of geraniol
GO: Geraniol
IL-1β: Interleukin-1β
MG: Methotrexate and high dose of geraniol
MMP-9: Matrix metalloproteinase-9
miRNAs: MicroRNAs
mTOR: Mammalian target of rapamycin
MX: Methotrexate
NF-kB: Nuclear factor kappa B
NLRP3: Inflammasome
NR: Healthy control rats
TNF-α: Tumor necrosis factor α
VEGF: Vascular endothelial growth factor
